# Upper Limb Tremors Classification for Parkinson’s Disease Using W-Band (76–81 GHz) Doppler Millimeter-Wave Sensing and Deep-Learning-Based Classifier

**DOI:** 10.3390/s26123955

**Published:** 2026-06-22

**Authors:** Pi-Yun Chen, Chun-Yu Lin, Neng-Sheng Pai, Ping-Tzan Huang, Chao-Lin Kuo, Chien-Ming Li, Chia-Hung Lin

**Affiliations:** 1 Department of Electrical Engineering, National Chin-Yi University of Technology, Taichung City 41170, Taiwan; qwertyuio890301@gmail.com (C.-Y.L.); pai@ncut.edu.tw (N.-S.P.); 2 Department of Biomechatronics Engineering, National Pingtung University of Science and Technology, Pingtung 91201, Taiwan; huangpt317@gmail.com; 3 Department of Maritime Information and Technology, National Kaohsiung University of Science and Technology, Kaohsiung City 80543, Taiwan; clkuo@nkust.edu.tw; 4 Infectious Disease Division of Internal Medicine Department, The Tainan Municipal Hospital, Tainan City 701, Taiwan; 235813cmli@gmail.com

**Keywords:** Parkinson’s disease, upper limb tremor, micro-Doppler effect, short-range and contactless, time–frequency transform, two-dimensional convolutional neural network

## Abstract

Parkinson’s disease (PD) is a neurodegenerative disorder with an increasing incidence rate that significantly affects patients’ motor functions and quality of life. Involuntary upper limb tremors (ULTs) commonly manifest unilaterally, affecting either the left or right upper limb. Clinically, ULT frequencies can be categorized into three distinct classes: low-frequency (<4.0 Hz), mid-frequency (4.0–7.0 Hz), and high-frequency (>7.0 Hz) tremors. These tremor motions are characterized by oscillatory or rotational (angular displacement) movements, commonly referred to as the micro-Doppler effect (mDE). This study aims to develop a short-range (<1.0 m) and contactless sensing method for ULT detection based on Doppler millimeter-wave (mm-Wave) radar. The reflected electromagnetic waves indicate time-varying frequency characteristics, which can be analyzed by using time–frequency transform (TFT) methods, such as the Wigner–Ville distribution (WVD) and smoothed pseudo WVD (SPWVD). These TFT methods are employed to extract mDE features, which are subsequently visualized as color-coded spectrograms for ULT classification. Then, a two-dimensional (2D) convolutional neural network (CNN) is employed to automatically recognize the visual feature patterns for ULTs classification based on frequency and amplitude information. In the experimental setup, the W-band (76–81 GHz) Doppler mm-Wave biosensor is implemented for sensing and extracting feature patterns. The proposed classifiers based on “WVD + 2D CNN” and “SPWVD + 2D CNN” are trained and validated by using the collected datasets, with 60% randomly selected for training datasets and 40% for testing datasets in each fold validation. A 10-fold cross-validation method is applied to evaluate the classifier’s performances, achieving an average precision of 95.92 ± 0.60%, average recall of 95.89 ± 0.62%, average F1-score of 0.9588 ± 0.0060, and average accuracy of 95.89 ± 0.62%, respectively. The experimental results demonstrate the feasibility of the proposed classifier for real-time ULTs classification in PD patients using short-range (<1.0 m) and contactless sensing.

## 1. Introduction

According to the Ministry of the Interior’s (MOI) statistics in 2023 years, Taiwan’s elderly population aged over 65 accounted for more than 20% of the total population, and the nation is expected to become a super-aged stage (more than 25%) by 2025 [1,2]. In aging societies, neurodegenerative disorders are becoming increasingly prevalent among the elderly population, including Parkinson’s disease (PD), Alzheimer’s disease (AZD), dementia, and cerebellar atrophy. These diseases are characterized by a chronic progression, leading to a gradual decline in neurological function. Among these diseases, PD and AZD have the higher prevalence. In PD, the dopamine deficiency combined with impaired cholinergic function results in an inadequate supply of dopamine required for regulating muscle activity, leading to various motor dysfunctions [3]. In Europe, there are approximately 1.2 million PD patients, with an estimated 60,000 new cases diagnosed annually. In the United States, approximately 1.0 million people are affected by PD, and nearly 90,000 new cases are diagnosed with PD each year [4,5].

The onset of PD typically occurs between the ages of 40 and 60, and its incidence increases with age, significantly impacting the patient’s quality of life. The PD is a neurodegenerative disorder that encompasses several types of movement disorders. Involuntary upper limb tremor (ULT) with different frequencies and amplitudes is one of the primary motor symptoms; and ULT frequencies are divided into three distinct classes: low-frequency (<4.0 Hz), mid-frequency (4.0–7.0 Hz), and high-frequency (>7.0 Hz) tremors. Parkinsonian tremor (PDT) and essential tremor (ET) are the primary tremor syndromes, encompassing various motor impairments and rhythmic oscillations that primarily affect either the left or right upper limb, including rest tremor (RT), postural tremor (PT), action tremor (AT), kinetic tremor (KT), physiological tremor, and pill-rolling tremor [6,7,8,9,10,11], and is sometimes accompanied by vocal modulation, affecting the vocal cords, respiratory muscles, and articulation, as well as mild gait abnormalities [12,13,14,15]. The PDT typically begins in one upper limb and may gradually extend to the head, lower limbs, trunk, and facial muscles. Clinically, PD is classified into five stages. In stages 1 to 3, patients with PD experience mild motor symptoms or develop walking and balance impairments, emphasizing the importance of early diagnosis and treatment. Selecting appropriate medications and rehabilitation strategies tailored to the specific type of neurodegenerative disease is crucial for effectively slowing its progression. Hence, the development of a digital-assisted quantification tool enables objective ULTs classification in patients with PD and facilitates self-care assessment.

Patients with PD usually exhibit some symptoms, such as ULTs, freezing of gait (FOG), bradykinesia, and dystonia [6,7,8,14,15,16,17,18,19,20]. Digital-assisted quantification tools have been utilized to detect FOGs and ULTs in the lower or upper limbs, respectively. The assistive tools for these two conditions include wearable sensors (accelerometers, gyroscopes, and pressure sensors), cameras or infrared sensors, millimeter-wave (mm-Wave) sensors [14,15,16,17], and inertial measurement unit (IMU) and electromyography (EMG) sensors [18,19,21]. The aforementioned assistive tools facilitate early detection, long-term continuous monitoring, and personalized rehabilitation for patients with PD. The measurement signals can be analyzed by using time-–frequency transform (TFT) methods, such as short-time Fourier transform (STFT) and fast Fourier transform (FFT), to extract tremor intensity and frequency for further quantifying ULT levels to identify the PD severity [18,20]. Then, machine-learning (ML) or deep-learning (DL)-based methods can be used to design a classifier for recognizing feature pattern for ULTs classification, including support vector machine (SVM), recurrent neural network (RNN), visual geometry group (VGG, University of Oxford) CNN (convolutional neural network) with 16 or 19 layers, and residual network (ResNet) with 18 or 50 convolutional (Conv.) layers (ResNet-18 or ResNet-50) [7,10,11,19,21,22,23,24]. However, the aforementioned assistive tools rely on contact-based sensor patches on the human body to measure the physiological signals, such as EMGs, electroencephalogram (EEGs), tremors, postures, and gaits [14,15,16,17,18,19,20,25,26]. These contact-based sensing methods need to collect large-scale measurement data for physiological signal analysis, statistical processing, and feature extraction and integrate with classifiers for quantifying severity levels and classes. In addition, the contact-based sensing methods also require prolonged monitoring, which may lead to drawbacks such as discomfort, increased stress, restricted movement, and low participant acceptance [7,10,11,17,20,21].

Contactless sensing methods, such as hand-drawn Archimedean spirals [10,11,23,24] and mm-Wave sensor [7,27,28,29], offer non-wearable, short-range, and non-contact sensing with personalized and repeatable operations. The spiral trajectories of the drawn patterns can be digitized, and these drawn patterns highlight distinct differences between normal individuals and patients with PD. For feature analysis, time-domain parameters, polar coordinate features, and frequency-domain parameters [11,12,30,31] facilitate the identification of nonlinear patterns associated with different tremor classes. In addition, the STFT method can be used to extract the time–frequency feature parameters. By leveraging color-encoded feature patterns associated with varying ULT intensities, ML- and DL-based classifiers can also be trained for automated tremor classification, including low-frequency tremor, PDT, and ET symptoms. However, the hand-drawing method must be correlated with the Movement Disorder Society’s Unified Parkinson’s Disease Rating Scale (MDS-UPDRS) assessment (0 to 4 scoring levels) [32,33] to define the severity levels and ULT classes. Thus, digital assistive tools, such as an iPad with Apple Pencil or ink pen or a touch screen [11,12,30,31], remain essential for efficient data collection and analysis. Additionally, the evaluation process is significantly time-consuming.

**Figure 1:**
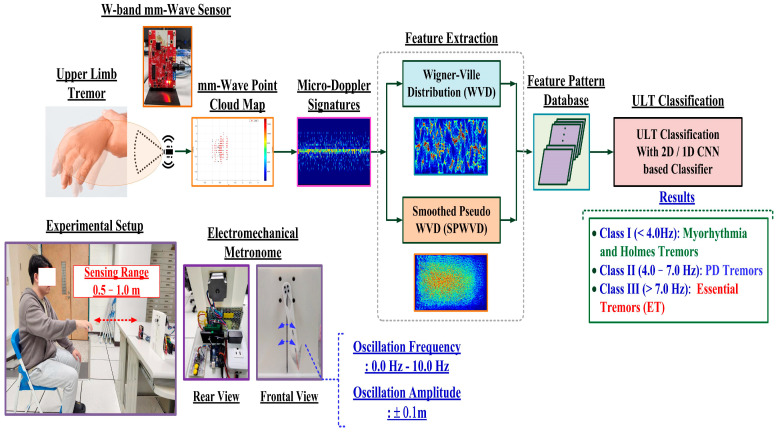
Configuration of W-band mm-Wave-based biosensor and experimental setup for ULT detection and classification.

The mm-Wave sensors (as seen in Figure 1), such as X-band (8–12 GHz), V-band (57–64 GHz), or W-band (76–81 GHz) frequencies, enable short-range and contactless detection for identifying and tracking the dynamic objects, including ULT, respiration, heartbeats, and body gesture monitoring [7,34,35,36,37,38,39]. Low-frequency X-band sensor is suited for longer range detection and better penetration through obstacles for big object movements but has lower resolution for detecting small and fine movements. Mid-frequency V-band sensors provide higher resolution and are more suitable for detecting subtle motions; however, they are more susceptible to atmospheric absorption. In short-range sensing applications, atmospheric absorption only slightly reduces the signal-to-noise ratio (SNR) during a single measurement and can therefore be neglected. The V-band sensor presented in [39] employs a 60 GHz frequency-modulated continuous-wave (FMCW) radar to measure displacement and velocity variations during sit-to-stand and stand-to-sit postural transitions in a residential environment. The extracted motion-related parameters are subsequently utilized as digital biomarkers for assessing pain conditions and physical decline. The high-frequency W-band sensor also provides the best resolution for precise motion tracking for small and fine movements in short-range sensing. The ULT movements can manifest as either regular or irregular oscillations with symmetrical or asymmetrical amplitudes, sometimes accompanied by subtle rotational motions, the so-called micro-Doppler effect (mDE). Hence, we select the W-band Doppler mm-Wave radar [37,38] to establish a short-range and contactless biosensor for detecting subtle upper limb displacements, which relies on reflected electromagnetic signal to analyze the object’s motions without physical contact. The object’s motions will result in reflected electromagnetic signals with time-varying velocity variations and frequency shifts, which can be represented as a point cloud map. Then, the TFT methods, such as Wigner–Ville distribution (WVD) and smoothed pseudo WVD (SPWVD) methods [40,41,42], are employed to extract the power spectrum from the time-domain micro-Doppler signatures (mDSs) and to generate the two-dimensional (2D) colored visualization feature patterns, as seen in Figure 1. The aforementioned WVD provide promising time–frequency resolution for analyzing nonlinear and time-varying frequency signals, while also preserving the total signal energy to represent the power distribution across both time and frequency domains. The SPWVD method applies a smoothing kernel window to the WVD process, thereby effectively reducing cross-term interference while preserving high-resolution feature characteristics. Both WVD and SPWVD can handle the time–frequency varying signals, making them suitable for real-time mm-Wave sensing applications and also significantly enhances the structural clarity of the tremor feature representations, thereby improving the discriminability of different ULT classes. Thus, the resulting feature patterns are fed into a one-dimensional (1D) and 2D convolutional neural networks (CNNs)-based classifiers to automatically perform the ULT identification across various tremor frequency ranges, as seen in Figure 1. The proposed classifier method is intended to provide a contactless ULT sensing and classification framework for assisting quantitative motion analysis, rather than serving as a standalone clinical detection tool for Parkinson’s disease.

The experimental setup was conducted in a laboratory environment (as seen in Figure 1), and the short-range (0.5 m–1.5 m) and contactless sensing framework was established by W-band mm-Wave-based biosensor (Texas Instruments (TI), Dallas, TX, USA). The proposed mm-Wave biosensor was placed on the desk in front of the enrolled subject with sensing range, 0.5–1.0 m, to sense the ULTs. An electromechanical metronome was employed to generate the driving force by using a pulse-width modulation (PWM) controller [7] and operated within an oscillation frequency range of 0.0 Hz to 10.0 Hz. Referring to the metronome, each enrolled subject was instructed to simulate ULT movements with rhythmic and symmetrical or asymmetrical oscillations at each sensing range, maintaining an oscillation amplitude of ±0.1 m. The oscillation frequencies were divided into three classes: Class I: low-frequency (<4.0 Hz), Class II: mid-frequency (4.0–7.0 Hz), and Class II I: high-frequency (>7.0 Hz). The raw mDSs of different ULTs were collected and processed for feature extraction with the WVD and SPWVD methods. Hence, the color-encoded feature patterns could be collected and be divided into training and testing datasets for training and validating the proposed classifier models, including “WVD + 1D CNN or 2D CNN classifier” and “SPWVD + 1D CNN or 2D CNN classifier”. A 10-fold cross-validation method was adopted, in which 60% of the dataset was randomly selected for training datasets and the remaining 40% was used as the testing datasets in each fold. The classifier’s performances were evaluated using precision (%), recall (%), F1 Score, and accuracy (%). The experimental results demonstrated the feasibility of the proposed classifier model and the most suitable sensing range for real-time ULT classification.

## 2. Methodology and Materials

### 2.1. Principles of Contactless W-Band (76–81 Ghz) Mm-Wave Based Biosensor

**Figure 2:**
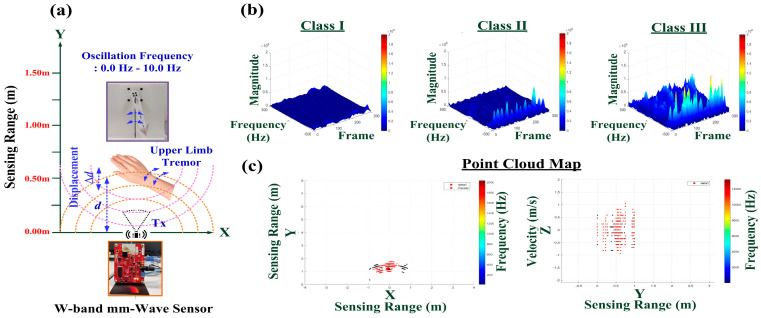
Short-range sensing experimental setup, mm-Wave signal processing, and feature parameters extraction. (**a**) Distance estimation between Tx and desired object, (**b**) Range- and Doppler-FFT processing for three classes, and (**c**) feature parameters extraction (X, Y, Z (m/sec)).

As seen in the experimental setup in Figure 1, the W-band mm-Wave firmware used is a 76–81 GHz mm-Wave-based Doppler radar with 2 Txs (2-transmit) and 4 Rxs (4-receive) phased array antennas, offering a 120° azimuth field of view (FoV) and a 30° elevation FoV and a substantial 4 GHz of continuous bandwidth (BW); and Rx noise figure is 14 dB for the 76–77 GHz and 15 dB for the 77–81 GHz. This hardware–software (HW–SW) device is an integrated single-chip mm-Wave sensor (ARM^®^ Cortex^®^-R4F-based radio control system) based on frequency-modulated continuous-wave (FMCW) radar platform, consisting of a mm-Wave sensor (IWR1642 BOOST# Evaluation Module, Texas Instruments, Dallas, TX, USA) and Python application software (Version 3.7). The proposed mm-Wave biosensor transmits electromagnetic waves via 2 Txs towards dynamic objects, which reflect the electromagnetic waves back to 4 Rxs, as seen in the configuration in Figure 2. The mm-Wave sensor with 2 Tx and 4 Rx antennas configuration is adopted to enhance the spatial resolution and provides angle-of-arrival (AoA) information. The multiple-input multiple-output (MIMO) virtual array structure significantly improves the capability to resolve fine-grained ULT motion signatures and suppress multipath interference, which is critical for reliable micro-Doppler (mD) feature extraction. After capturing and preprocessing the reflected electromagnetic signals, the displacement (amplitude) and velocity (frequency) changes caused by the objects can be detected, allowing for estimating the object’s movement state, distance, velocity, direction, or angle. Assuming the transmitted electromagnetic signal is *s*(*t*) [7]

(1) $$s(t)=A_{0}\cos(2\pi f_{0}t)$$

where *A*_0_ is the signal amplitude; *f*_0_ is the signal frequency. Given *f_T_* and *θ*(*t*) as the transmitted frequency and phase noise, respectively, the reflected electromagnetic signal, *R*(*t*), can be represented as [7]

(2) $$R(t)\approx A_{R}\cos(2\pi f_{T}t)-\frac{4\pi d_{0}}{\lambda_{T}}-\frac{4\pi x(t)}{\lambda_{T}}+\theta (t-\frac{2d_{0}}{c})$$

(3) $$x(t)=A_{1}\cos(2\pi f_{1}t)$$

where *A*_R_ is the amplitude of the reflected signal, *R*(*t*); *λ_T_* = *c*/*f_T_* is the wavelength (*c* = 3 × 10^8^ m/s); and *d*_0_ = *ct_r_*/2 is the distance between the object and the Txs; *t_r_* is a delay time on the time axis. For ULT detection, *x*(*t*) is the ULT’s dynamic movement signal, *f*_1_ is the tremor oscillation frequency (irregular or regular) and *A*_1_ is the tremor oscillation amplitude (symmetrical or asymmetrical). Assuming *θ*_0_ = 4*πd*_0_/*λ*_T_ as the phase shift and Δ*θ*(*t*) = *θ*(*t*) − *θ*(*t* − 2*d*_0_/*c*) as the residual phase noise, the normalized baseband signal, *B*(*t*), can be obtained through the mixing and low-pass filtering processes, and then an intermediate frequency (IF) signal is generated, which contains crucial information, including the object’s angle, distance, and velocity [7]

(4) $$B(t)\approx \cos(\theta_{0}+\frac{4\pi x(t)}{\lambda_{T}}+\Delta \theta (t))$$

Considering phase angle, *θ*_0_, as an odd multiple of *π*/2 and *s*(*t*) *<< λ_T_*, Equation (4) can be simplified to the general mathematical form as Equation (5):

(5) $$B(t)\approx \frac{4\pi x(t)}{\lambda_{T}}+\Delta \theta (t),\;x(t)\approx d_{0}\pm \Delta d(t)$$

where Δ*d*(*t*) is the object’s variation range with regular or irregular motions. If phased array antennas are used, the synthesized signal for multi channels is

(6) $$x_{T}(t)=\sum_{i=1}^{T}B_{i}(t),i=1,2,\ldots ,T(T=4\mathrm{in}\mathrm{this}\mathrm{study})$$

The synthesized signal, *x_T_*(*t*), can be processed through an analog-to-digital converter (ADC), which then sends the digital raw data to an embedded system or a laptop. After digital filtering removes unwanted high-frequency and low-frequency components, the objects’ displacement and velocity changes are estimated through Range-FFT and Doppler-FFT processes.

Before digital mm-Wave signal processing, the sensing data stream can be stored in an N × M matrix, where *M* represents the number of chirps and N represents the number of samples per chirp signal. In mm-Wave signal processing, each chirp signal is sequentially read and processed through the Range-FFT and Doppler-FFT processes, target searching, phase extraction, phase unwrapping, phase difference operation, and filtering processes, as seen in Figure 2. The two key signal processing methods, Range-FFT and Doppler-FFT operations, are used to estimate the range, displacement, and velocity changes of the detected potential objects. The two operations are described as follows:

- Range-FFT processing: The synthesized IF signal, *x_T_*(*t*), is processed using a 1D FFT to extract the distance information between the desired object and the Tx and then produces a time delay *τ* (sec) between them and results in a frequency tone, *S_τ_*. By performing Range-FFT operation, the frequency response of the chirp signal (the signal *x_T_*(*t*) in Equation (6)) can be estimated for finding out the peak value of the frequency spectrum (as seen in Range- and Doppler-FFT processing for three classes in Figure 2b). Then, the distance (location), *d*, between the desired object and the Tx can be determined (as seen in distance estimation in Figure 2a) by general form as

(7) $$d=\frac{cT_{c}}{2B}S_{\tau }(m),\;S=\frac{B}{T_{c}},\;S_{\tau }=S\tau$$

where *c* = 3 × 10^8^ (m/s) is the velocity of electromagnetic waves in the air; *B* is the bandwidth; *T_c_* is the timing duration; and *S* is the slope of the chirp signal.

**Figure 3:**
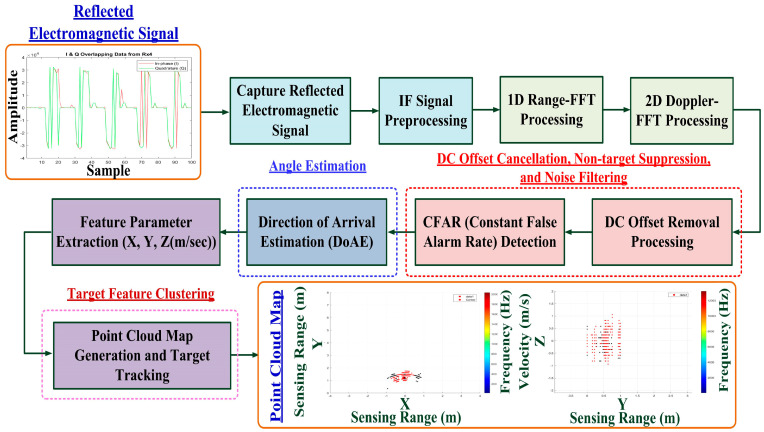
Flowchart of mm-Wave signal processing and feature parameter extraction (Point Cloud Map Generation).

- Doppler-FFT process: Capturing *M* chirps results in *M* phases and treating these *M* phases as a signal and performing an FFT operation, the velocity of the desired object can be estimated by using the peak values obtained from every two phases as follows:

(8) $$v=\frac{\lambda \Delta \theta }{4\pi T_{c}}\;(m/\sec),\;\Delta \theta <\pi$$

where phase differences, Δ*θ*, can be obtained through the Direction of Arrival Estimation (DoAE) and Angle Estimation (as seen the flowchart in Figure 3); and *λ* is the wavelength of electromagnetic wave. Hence, the 1D Range-FFT operation can be used to estimate the distance of the possible desired object; thus, the objects’ dynamical displacement information can be obtained. The 2D Doppler-FFT operation can detect the velocity of the desired object motions. As seen in the flowchart in Figure 3, this study employs the 1D Range-FFT operation to estimate the displacement variations caused by symmetrical or asymmetrical ULTs; after the Range-FFT processing, the 2D Doppler-FFT is applied to the consecutive frames (chirp sequences) along the time axis to obtain the object’s moving velocity information, as seen in the point cloud map (X, Y, Z (m/sec)) in Figure 2c.

### 2.2. Micro-Doppler Feature Extraction and Enhancement

The ULT refers to irregular or regular oscillatory motion and is characterized by symmetrical or asymmetrical amplitudes, as well as rotational or vibratory components in upper limb movements. These subtle motions cause small but distinguishable frequency shifts superimposed on the reflected signals, resulting in unique mD feature patterns. With the 300-frame (33.33 ms per frame) data acquisition window lengths for framing processes, the mD feature patterns can be extracted from point cloud map, as seen in the feature patterns for three ULT classes in Table 1, which can be used to distinguish the mD feature patterns among three ULT classes related to different tremor frequency, oscillation and/or rotational motions. For the time–frequency analysis, the WVD- and SPWVD-based TFT methods can enhance the resolutions of mD feature pattern for refined color visual representations and then generate the 2D color-encoded feature patterns, as seen in the 875 × 656 pixel patterns in Table 1. Theses TFT methods are used to analyze the variations in mD feature patterns in both time and frequency domains. Compared with STFT method, the WVD method provides higher time–frequency resolution by applying the Fourier transform to the time-dependent autocorrelation function, *R*(*t*,*τ*), and the power spectrum is transformed into a time-dependent function, “*R*(*t*,*τ*)● exp(−*j*2*πfτ*)”, allowing the frequency parameters of the signal *x_T_*(*t*) to be extracted within a specific time range, including instantaneous frequency, frequency centroid, and energy distribution. The extracted feature patterns can be transformed into a color-encoded representation, as shown in Table 1. The general formula of WVD for signal *x_T_*(*t*) is defined as [40,41,42]:

(9) $$\mathrm{WVD}(t,f)=\int_{-\infty }^{+\infty }z(t+\frac{\tau }{2})z*(t-\frac{\tau }{2})e^{-j2\pi f\tau }d\tau ,\;R(t,\tau )=z(t+\frac{\tau }{2})z*(t-\frac{\tau }{2})$$

(10) $$z(t)=x_{T}(t)+jH[x_{T}(t)]$$

where *τ is* the timing shift; *ω* = 2*πf* and *f a* are frequency; z*(●) is the complex conjugate operation; and *H*[●] is the Hilbert transform operation for signal *x_T_*(*t*). The Fourier transform of the time-dependent autocorrelation function can by computed by integral operation. However, while signal contains multiple frequency components, the WVD has cross-term interferences, leading to spectral interferences, readability decreasing, and negative energy artifacts in the spectrogram, and will affect the clarity of time–frequency feature patterns. Hence, the SPWVD method can reduce aforementioned interferences through time and frequency smoothing processes. The general formula is defined as [40,41,42]:

(11) $$SPWVD(t,f)=\int_{-\infty }^{+\infty }\int_{-\infty }^{+\infty }g(\tau )h(f)x_{T}(t+\frac{\tau }{2})x_{T}^{*}(t-\frac{\tau }{2})e^{-j2\pi f\tau }d\tau df$$

where *g*(*τ*) is the smoothing window along the time-axis and *h*(*f*) is the smoothing window in the frequency direction. In this study, “Hamming window” is chosen as both the time and frequency domain windows, which are used to reduce time-domain interference and to suppress cross-terms in the frequency domain. Hence, it can reduce the cross-term interferences while also maintaining the time–frequency features and enhancing resolution, thereby improving the distinguishability among different ULT classes.

**Figure 4:**
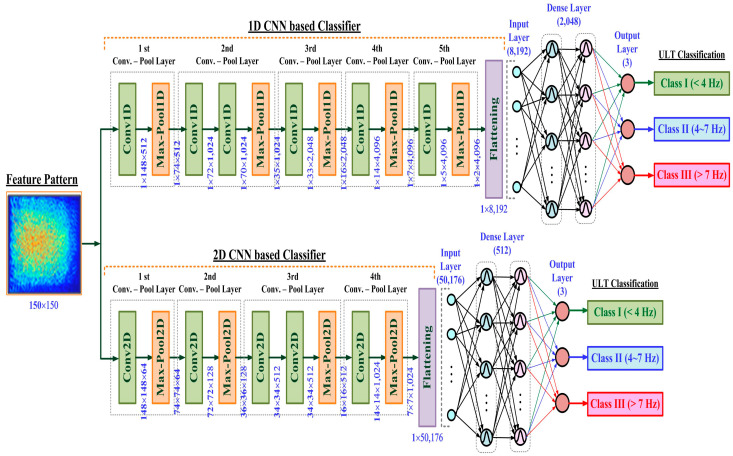
Structure of the proposed 1D CNN and 2D CNN based classifiers for ULT classification.

For feature extraction, this study employs the WVD- and SPWVD-based TFT methods to extract feature patterns of different ULT motions as pathological oscillation frequencies in three ranges: Class I: <4.0 Hz; Class II: 4.0–7.0 Hz; and Class III: >7.0 Hz, along with their corresponding possible ULT classes, including Myorhythmia and Holmes tremors, PDT (RT and PT), and ET (PT and KT) [7,30], as seen in Table 1. Experimental setup was conducted in our university laboratory (Micro-processor Laboratory, National Chin-Yi University of Technology (NCYUT), Taichung City, as seen in Figure 1), and each enrolled subject was asked to sit on a chair in front of a desk, while the mm-Wave sensor was positioned facing the subject at sensing ranges from 0.5 m to 1.5 m. The mm-Wave sensor was placed at the same height as the subject’s hand, maintaining approximately 0.6 m above the ground. A metronome was placed on a table at a height of approximately 0.5 m to provide reference oscillation frequencies during the experiments. During each measurement, enrolled subjects were required to sit upright on a chair, with their upper body and lower limbs kept stationary and no voluntary movements allowed. Following the metronome reference frequencies ranging from 0.0 Hz to 10.0 Hz, each subject was instructed to perform left-to-right hand-swing motions to simulate ULT movements, including regular and irregular ULT movements, as well as symmetric and asymmetric amplitude characteristics, as shown in Figure 2a. The experiments were conducted at three different sensing ranges of 0.50 m, 1.00 m, and 1.50 m, respectively. The hand-swing displacement was maintained at approximately 0.15 m during each measurement. For two repeated measurements at each visit site, raw datasets were collected under resting conditions and three ULT classes. Each measurement yielded more than approximately 300 frames (approximately 10 s, 33.33 ms per frame). The measurement data were subsequently transmitted to a PC via Universal Asynchronous Receiver/Transmitter (UART) for storage and further analysis. In the preprocessing stage, manual labeling was performed to annotate tremor frequency and sensing range for each sample, which was used to construct the raw dataset for classifier development. The feature patterns were extracted from collected raw datasets (as seen in velocity versus time mD feature patterns in Table 1) by using WVD and SPWVD methods, as seen in frequency versus time feature patterns in Table 1, which could be divided into training datasets and testing datasets, as seen in Table 2. Subsequently, these datasets were used to train and validate the “WVD + 2D CNN”, “WVD + 1D CNN”, “SPWVD + 2D CNN”, and “SPWVD + 1D CNN” based classifiers, as seen in the structures in Figure 4.

### 2.3. Pattern Classifier Design

The small-scale model of cascade CNN (CCNN) was used to establish the 2D CNN and 1D CNN based classifiers, including “WVD + 2D CNN”, “WVD + 1D CNN”, “SPWVD + 2D CNN”, and “SPWVD + 1D CNN”. The aforementioned classifiers can perform the pattern recognition tasks for ULTs classification. The optimal classifier model, feature representations, and measurement sites are also evaluated, along with the applicability of the proposed DL-based model for the intended application. These CCNNs consist of multi convolutional–pooling (Conv.–Pool) layers, one flattening layer, and a fully connected layer (classification layer) with multi dense networks [21,22,23,38], which can be trained to automatically enhance features, extract features, and perform the pattern recognition tasks. Through multi Conv. operations with multi sliding 3 × 3 kernel windows, the multi Conv.–Pool layers can obtain a weighted linear combination of convolution kernels to raise both the depth and width levels of feature patterns. The multi Conv. operations can also increase the dimensionality, non-linearity, and complexity of feature patterns, thereby enhancing the classifier’s ability to recognize complex feature patterns. By applying the maximum pooling (Max-Pool) operations, these processes can reduce the size of the feature patterns and can also maintain the key feature parameters while preserving the depth level of the feature patterns. The 4 2D Conv.–Pool layers and 5 1D Conv.–Pool layers are designed for 2D and 1D CNN based classifiers, respectively, as seen in Figure 4. Hence, the CCNN can directly extract features from 2D color-encoded feature patterns and resize the input pattern size from 875 × 656 pixels to 150 × 150 pixels for training and validating the classifiers. The fully connected layer consists of a flattening layer, multi dense networks, and an output layer. In each dense network, every neuron node is connected to all neuron nodes in the preceding and succeeding layers, allowing it to receive inputs from all neuron nodes in the previous layer. The output *y* of each neuron node can be computed by the general formula [38,43,44]

(12) y(W,x)=f(Wx+b)

where *x* is the input feature vector; *W* is weighted matrix in each dense network; *b* is the bias weight; and function *f*(●) is the activation function, and the Gaussian error linear unit (GeLU) function is used instead of traditional rectified linear unit (ReLU) function, which can mitigate the problem of gradient vanishing [38,44,45]. The dense networks are descending powers based on the number of input data and output classes (3 Classes), and their relevant design parameters for 2D CNN and 1D CNN based classifiers are depicted in Figure 4.

For multi-class classification problems, the categorical cross-entropy (CCE)-based loss function (*LF*) is employed to evaluate the performance of classifier training, which measures the dissimilarity between the target labels and the predicted probability distribution output by a classifier. The CCE general formula is defined as [38,43,44]

(13) $$\mathrm{LF}=\sum_{k=1}^{K}\sum_{j=1}^{C}y_{k,j}\log({y^{\prime }}_{k,j})$$

where *y_k_*_,*j*_ is the target label with the “one-hot encoded” as [1, 0, 0], [0, 1, 0], and [0, 0, 1] for three classes, respectively; *y*’*_k_*_,*j*_ is the predicted probability of *j*th class (*C* = 3) for *k*th training dataset (*k* = 1, 2, 3, …, *K*). The logarithm operation, log(●), can ensure that incorrect predictions are penalized more severely. The Softmax activation function is used in the final output layer for multi classes; the Softmax general formula is defined as [44,45]

(14) $${y^{\prime }}_{j}=\frac{\exp(z_{j})}{\sum_{j=1}^{3}\exp(z_{j})},\;j=1,\;2,\;3$$

where *z_j_* is the raw logits for *j*th class, as output vector *Z* = [*z*_1_, *z*_2_, *z*_3_], and exp(*z_j_*) operator exponentiates the logits to make them a positive value. The higher logits lead to higher probabilities, and the highest probability class is the possible classifier’s prediction. The optimization algorithm, such as adaptive moment estimation (ADAM) optimizer, is employed to train the 2D CNN and 1D CNN classifier models to minimize the CCE LF. It utilizes iterative computations with smoothing and adaptive updates and an adjustable learning rate to enhance training efficiency. The mean and variance of gradients with bias-corrected estimates are used to update the classifier’s parameters for mitigating the vanishing and exploding gradient problems. Finally, the 10-fold cross-validation method is applied to evaluate the classifier’s performance. The feasibility of the proposed classifier is assessed by using precision (%), recall (%), F1 Score, and accuracy (%) as evaluation metrics [38,43].

## 3. Experimental Results and Discussion

### 3.1. Case Study 1: Classifier Training and Validation in a Fixed Measurement Site

**Figure 5:**
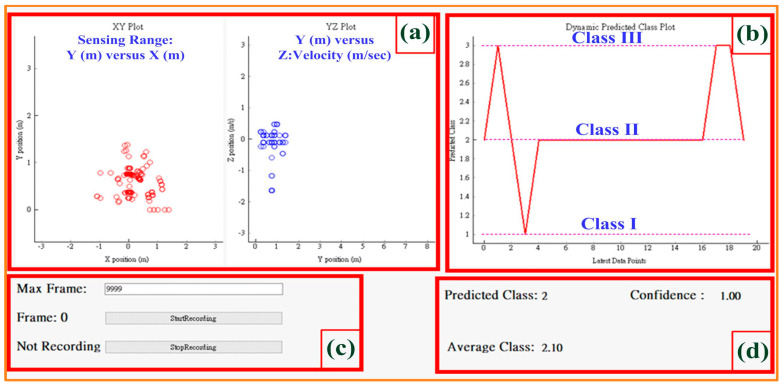
Graphical user interface (GUI) for data visualization and biosensor system monitoring. (**a**) ULT point-cloud visualization map (Class II: 4.0–7.0 Hz), (**b**) ULT screening results, (**c**) operational parameter control, (**d**) classification outputs accompanied by confidence indicators.

Experiments were conducted in a university laboratory environment, as seen in the Micro-processor Laboratory in Figure 1. Ten enrolled subjects (gender: male subject; age: 22.5 ± 2.5 years old) were invited, and each enrolled subject was asked to sit on a chair in front of a desk, with each participant undergoing two test sessions. The W-band mm-Wave-based biosensor was placed at different measurement sites, as sensing ranges from 0.50 m to 1.50 m, as seen in Figure 2a. Measurements were taken with data acquisition lengths of 300 sequential frames (each experiment lasted approximately 10 s) at three different locations (0.50 m, 1.00 m, and 1.50 m). Referring to the metronome with varying 0.0 Hz to 10.0 Hz oscillation frequencies, each enrolled subject simulated regular or irregular ULT motion with symmetrical or asymmetrical amplitudes in three ranges of oscillation frequencies: (1) <4.0 Hz, (2) 4.0–7.0 Hz, and (3) >7.0 Hz. Practical radar measurements were conducted using the experimental setup, as shown in Figure 2a. To support the real-time measurements and mm-Wave sensing operations, a graphical user interface (GUI) was developed for data visualization and biosensor system monitoring. As seen in GUI in Figure 5, the GUI consists of four functional modules: (a) real-time ULT point-cloud visualization map (with Class II: 4.0–7.0 Hz tremor used as an example); (b) ULT screening result display; (c) operational parameter control; and (d) classification outputs accompanied by confidence indicators. This interface enabled intuitive system monitoring and facilitated efficient ULT screening (Class I~Class III) and result interpretation. As shown in Figure 5a, the ULT point-cloud visualization consisted of both XY (sensing range: Y (m) versus X (m)) and YZ (Y (m) versus X: velocity (m/sec)) plane feature maps, which were used to display the spatial distribution of reflection points detected by the mm-Wave biosensor in real time. This visualization function enabled ULT to be intuitively represented and observed, thereby facilitating real-time assessment of motion distribution and stability within the sensing region.

The numbers of collected raw measurement data at different measurement sites (0.5 m, 1.0 m, and 1.5 m) from 10 enrolled subjects for three classes were presented in Table 2. A total of 3000 raw datasets were collected and could be divided into 1800 for training datasets and 1200 for testing datasets at each measurement site (60% for model training and 40% for model testing), respectively. During the classifier model training and validation stages, subject-level data separation was strictly enforced to prevent samples from the same subject from appearing in both the training and testing datasets, thereby mitigating the risk of data leakage. The “mD feature pattern + WVD” and “mD feature pattern + SPWVD” were used to extract the colored visualization feature patterns (as shown in Table 1) for trained 2D- and 1D-CNN-based classifiers. This study designed CCNN architectures to realize pattern-recognition classifiers, as seen in Figure 4. Different CCNN architectures were implemented in a computer-based platform (Intel^®^ Q370, Intel^®^ Core™ i7 8700, DDR4 2400 MHz 8G*3) and also used the graphics processing unit (GPU: NVIDIA^®^ GeForce^®^ RTX™ 2080 Ti, 1755 MHz, 11 GB GDDR6) [46] to speed up the execution time for ULTs classification. For example, in Figure 4, as 2D CNN, first two Conv.–Pool layers were used to extract the initial feature parameters and then, second, two Conv.–Pool layers were employed to refine the extracted features and remove redundant information (from 148 × 148 × 64 to 7 × 7 × 1024). The dense network (50,176 × 512 × 3) performed the classification tasks based on high-level refined feature patterns.

**Figure 6:**
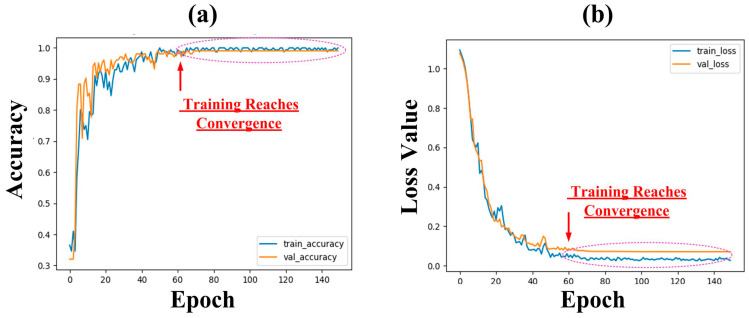
Training and validation history curves (train: blue line, val: orange line). (**a**) History curve of accuracy versus epoch; (**b**) history curve of loss value versus epoch.

The ADAM optimizer’s learning rate, momentum decay, and variance decay were set as 0.001, 0.900, and 0.999 (experience values) [38,43] and the maximum number of training iterations was set as 150 epochs. An iteration computational manner was employed to adjust the network parameters. For each ULT Class of datasets, 60% of training datasets were randomly used for classifier training, distributed as follows: Class I: 600, Class II: 600, and Class III: 600, and the remaining 40% for classifier testing, distributed as follows: Class I: 400, Class II: 400, and Class III: 400. Then, the ADAM optimizer was used to train the 2D and 1D CNN-based classifiers with 300 training datasets. The 200 testing datasets were utilized to evaluate the classifier’s recognition performance. We conducted a case study to train and validate the proposed classifiers in a fixed 0.50 m measurement site. This experimental setup ensured consistency in data acquisition, enabling a reliable evaluation for the classifier’s performances. For example, for “SPWVD + 2D CNN” based classifier, Figure 6a,b shows the training (train: blue line) and validation (val: orange line) history curves for accuracy saturation curves and convergence history curves, respectively. As the iteration numbers increased, the classifier’s output accuracy smoothly increased; conversely, the LF value gradually decreased to the specific convergence condition, and the val_loss followed train_loss without large divergence, which reached saturation state after 60 training epochs. Hence, with the 10-fold cross-validation, an average accuracy (%) of 95.89 ± 0.62% and an average loss value of 0.1244 ± 0.0012 were obtained after approximately 60 epochs. As seen in the testing results in Table 3, for the fixed 0.50 m measurement site, the 10-fold cross-validation results demonstrated that the performance of “SPWVD + 2D CNN” was better than the other three classifier models (“WVD + 2D CNN”, “WVD + 1D CNN”, and “SPWVD + 1D CNN”), as the average F1 score was greater than 0.9000 (approaching 1, indicating a promising classifier that effectively balanced precision and recall), and average accuracy (%) was also higher than other 2D and 1D CNN models. Hence, Case Study#1 suggested the following: (1) the promising classifier model could be determined by combining SPWVD-based feature extractor and 2D CNN, as seen in the grey highlights in Table 3; (2) 2D CNN was effective for 2D spatial patterns, such as time–frequency spectrograms; and (3) 2D Conv. operations increased the dimensionality and non-linearity of feature patterns to enhance the ability to recognize the complexity feature patterns.

### 3.2. Case Study 2: Classifier Validation in Different Measurement Sites

**Figure 7:**
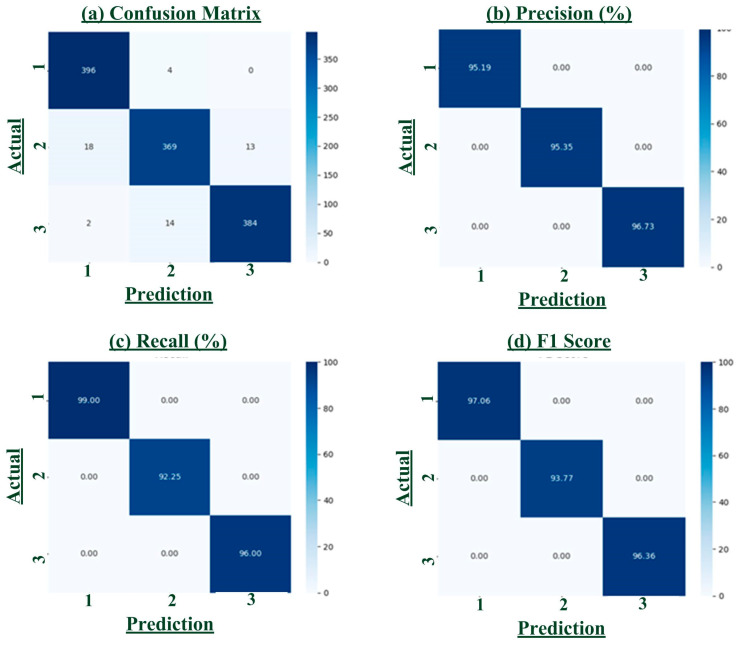
Output confusion matrix of the best-performing classifier model “SPWVD + 2D CNN”. (**a**) Normalized confusion matrix for the three-class classification, (**b**–**d**) precision (%), recall (%), and F1-score for the three-class classification.

To validate the feasibility of short-range continuous monitoring, we asked each enrolled subject to place the right or left upper limb at three locations, 0.50 m, 1.00 m, and 1.50 m under resting condition, as seen in Figure 2a. For the same experimental setup, referring to the metronome, each enrolled subject was instructed to maintain a stationary upper limb and to simulate ULTs with symmetrical ± 0.1 m oscillation amplitudes and different pathological frequencies at different measurement sites, including Class I: <4.0 Hz, Class II: 4.0–7.0 Hz, and Class III: >7.0 Hz. The raw sensing ULT signals could also be collected for signal preprocessing and feature extraction with WVD and SPWVD. In an additional 6000 experimental tests at locations 1.00 and 1.50 m (as seen Table 2), the training and testing datasets were also prepared for training four classifier models, as seen in Table 4. We also evaluated the different classifiers’ performances at different measurement sites by using the F1 score (the harmonic mean of precision and recall) and accuracy (%) indexes. With the 10-fold cross-validation, the average F1 score and average accuracy (%) at different sensing ranges for four classifier models are shown in Table 4. The key feature parameters gradually diminished as the sensing range increased from 0.50 m to 1.50 m, due to the attenuation of the radar microwave signal intensity. Therefore, the attenuated feature patterns would affect the classifier’s pattern recognition capability. Two key evaluation indexes decreased as the sensing ranges increased for the aforementioned four classifiers. It could be seen that the promising measurement site at “*location 0.50 m*” for ULTs monitoring and classification with “SPWVD + 2D CNN” and “WVD + 2D CNN” based classifiers. The average F1 scores for both classifiers were 0.9588 ± 0.0060 and were greater than 0.9000, meaning that classifier had few false positives and false negatives and underscoring the classifiers’ reliability in differentiating the three tremor classes. The average accuracy (%) was 95.89 ± 0.62% for identifying the tremor classes. Overall testing results for four classifier models are shown in Table 4. Taking the best-performing classifier model “SPWVD + 2D CNN” as an example, the normalized confusion matrix for the three-class classification task is shown in Figure 6a. The classifier’s precision (%), recall (%), and F1-score for the three-class classification are also shown in Figure 7b to d, respectively.

For the same datasets and measurement site at location 0.50 m, with the 10-fold cross-validation, “SPWVD + 1D CNN “ had average precision (%) of 88.25 ± 1.39%, average recall (%) of 88.21 ± 1.37%, average of F1 score of 0.8816 ± 0.0130, and average accuracy (%) of 88.21 ± 1.37% for ULTs classification, and the experimental results of “WVD + 1D CNN” were better than the aforementioned 1D CNN model [47,48]. In addition, an ablation study was also conducted to evaluate the classifier’s performance. The feature-enhanced WVD and SPWVD methods were removed, and the 0.5 m measured testing datasets were directly applied to evaluate the “mD Feature Pattern + 2D CNN” classifier for ULT classification. This classifier model directly extracted mD feature patterns from the phase variations sensed by using our proposed mm-Wave sensor for ULT classification. The experimental results achieved an average precision (%) of 88.61 ± 1.37%, average recall (%) of 87.64 ± 1.41%, average F1 score of 0.8754 ± 0.0143, and average accuracy (%) of 87.65 ± 1.41%. For overall experimental results, it could be seen that the 2D CNN models outperformed the 1D CNN models across different measurement sites. Hence, Case Study#2 suggested the following: (1) the promising measurement site could be set at location 0.5 m, and the desktop sensing tool was feasible for a short-range and contactless continuous monitoring; (2) feature extraction from time–frequency spectrogram based on 2D spatial dimensions was beneficial for pattern recognition; (3) multi Conv.–Pool operations with multi Conv. kernel windows were used to establish a CCNN for progressively capturing both fine and coarse features to improve accuracy in pattern recognition tasks; (4) multi Conv. layers in a sequential manner could filter out irrelevant information, enabling a progressive learning approach that improved robustness to variations in input feature patterns.

### 3.3. Comparison with Existing Non-Contact and Short-Range Sensing Methods

Table 5 indicates the existing short-range and non-contact sensing methods that can continuously quantify tremor classes in a desktop setting without compromising the privacy of the enrolled subjects, such as digital handwriting analysis with an iPad (Touchscreen) and Apple Pencil [10,11,22,23], as well as Doppler mm-Wave biosensors with the X- (10.525 GHZ), K- (24 GHz), and W-band (76–81 GHz) radars [7,29,49,50]. Digital handwriting analysis was a non-invasive and easy-to-use tool that could be utilized in both hospital and home settings. In contrast to conventional paper-based handwriting tests, digital methods provided quantifiable measurements on pattern shapes, writing speeds, stroke pressures, and tremor frequencies. Referring to various predefined geometric patterns, such as spirals and straight lines (horizontal or vertical) [10,11], the aforementioned records could be transformed into a polar representation, including radius, angle, and tremor oscillations in a curved trajectory, to quantify different hand movement disorders for PD screening or early diagnosis. Then, ML- or DL-based classifiers, such as nonlinear SVM [10], General Regression Neural Network (GRNN) [11], VGGs [22], and 2D CNN [23], were applied for automatically analyzing the aforementioned feature patterns for subsequent recognition in PD screening, which indicated a mean accuracy (mean hit rate) of >90% for the intended purpose, as seen in the experiment results in Table 5. However, its sensing manner was assistive tool dependency, which required specialized hardware to access these digital handwritten patterns. Thus, older subjects or those unfamiliar with the digital assistive tool might have difficulty performing the handwriting tests. In addition, with variations in individual handwriting habits, stress levels, and medication intake, there was no gold standardized protocol for tremor quantification, potentially affecting the reliability of tremor analysis.

The mm-Wave sensing with FMCW were also non-contact, privacy-preserving, and real-time processing methods for continuous monitoring in ULT quantification [7,29,46,50], gesture recognition (wipe, swing, push, invalid, and circle), and gait symmetry analysis [51] applications. Their method was less affected by environmental factors compared to cameras or optical techniques. While frequency radar bands, such as 10 GHz, 24 HGZ, and 77 GHz, provided extended sensing ranges (<1.0, 3.0–6.0 m, or 0.0–10.0 m) and balanced resolution for general hand motion tracking, tremor detection, and gait analysis; their limited resolution reduced the sensitivity to mDE, thereby requiring a trade-off between sensing ranges and resolutions. For example, using X-band mm-Wave sensing with FMCW method, Reference [7] demonstrated good resolution at a measurement distance of 0.30 ± 0.1 m for time-domain parametric feature extraction. Three quantitative features, such as ZC, WAMP, and WL, showed a positive correlation (mean coefficient of determination, R^2^ > 0.85) with different ULT classes, which were effective in quantifying normal conditions, Myorhythmia and Holmes tremors (<4.0 Hz), and PDT (4.0–8.0 Hz). References [49,51] employed 24 GHz FMCW MIMO radar for non-contact posture recognition and gait symmetry analysis applications, respectively. Reference [49] applied beamforming CFAR detection and feature extraction methods to detect four hand gestures within a sensing range of <1.0 m, achieving a recognition accuracy of 99.60% for all gesture types. Reference [51] also employed the same 24 GHz radar to evaluate gait symmetry in early-stage PD within a sensing range of 0.0–10.0 m. Lower-limb gait features were extracted from the radar measurements, and the foot identification accuracy as well as gait asymmetry parameters were analyzed. For gait parameters, including step time, gait velocity, stride distance, and foot velocity, the average absolute error of the symmetry rate was reported to be less than 8%. In Reference [52], a distributed sensing network consisting of three orthogonally arranged 24 GHz Doppler radars was employed to measure the three-dimensional (3D) displacement of moving objects. A curve extraction algorithm was applied to separate different body parts, and the 3D displacement trajectories were subsequently estimated through temporal integration. A stereophotogrammetric system was further utilized as the ground-truth reference for performance validation. Experimental results obtained from human walking trails demonstrated that mD analysis could overcome the limitations of conventional phase-based methods in biomechanical applications. However, this 3D sensing framework required multiple radar units and a complex distributed deployment, resulting in increased system complexity and installation cost requirements. Reference [29] also demonstrated the use of a W-band (77.0 GHz) FMCW radar sensor for PDT and ET quantification. The tremor frequencies and amplitudes were extracted from reflected electromagnetic signals. The linear regression method was used to analyze the feature parameters obtained from the contactless and ground truth (accelerometer and gyroscope) sensing methods. Thus, compared to the ground truth method, the contactless mm-Wave sensing method demonstrated a high level of agreement (R^2^ > 0.97), indicating strong correlation for continuous monitoring to predict the level of tremor severity.

Therefore, the higher-frequency W-band radar had higher resolution mDE detection for maintaining sufficient precision in fine tremor quantification. In our study, we proposed the WVD- and SPWVD-based feature extraction methods [40,41,42] to enhance the mD signatures for improving recognition accuracy. For large hand motions, in Reference [49], the K-band mm-Wave radar had a balance between resolution, sensing range, and penetration for gesture recognition. The gestures’ 2D time–feature maps with a lightweight CNN were used to classify different hand gestures, achieving a recognition accuracy of 99.60% for five gesture classes, including wipe, swing, push, invalid, and circle. In our study, we proposed “WVD + 2D CNN” and “SPWVD + 2D CNN” based classifiers for automatic ULT classification and obtained promising average accuracies of 93.81 ± 1.88% and 95.89 ± 0.62% and average F1 scores of 0.9379 ± 0.1900 and 0.9588 ± 0.0060 for the intended purpose, respectively, as seen in Table 5. The W-band mm-Wave radar offered superior resolution compared to that of X- and K-band in tremor motions detection and detailed feature extraction. Hence, the proposed short-range and contactless sensing tool and classifier model had promising performances for real-time tremors monitoring and ULT classification.

## 4. Conclusions

Clinical assessments commonly relied on the MDS-UPDRS and Health-Related Quality of Life (HRQOL) [33,34] questionnaires for face-to-face evaluations. During PD examinations, physicians also observed abnormal symptoms based on the PD patients’ motor function, coordination, and balance, including whether the upper limb exhibited RT, muscle rigidity and bradykinesia during body movement, and the voice became softer. These symptoms would manifest in the upper or lower limbs, body, and vocal characteristics. However, these examination manners required significant time and human resources for questioning and scoring (from 0 to 4 scores). This traditional assessment method lacked scientific data quantification, automated detection, repeatability, and personalized application, posing limitations for standardized evaluations. Hence, we had proposed a short-range and contactless sensing method for ULT classification by using W-band mm-Wave biosensor, including three ranges of tremor frequencies and possible ULT classes, referring to low-frequency tremors (<4.0 Hz), PDT (4.0–7.0 Hz), and ET (>7.0 Hz). The WVD and SPWVD methods were employed to enhance the mD feature resolution, providing more refined and color-encoded feature representations for pattern recognition tasks. Considering trade-off among resolution, sensing range, and classifier model, we suggested the suitable measurement site at location 0.5 m and designed the “SPWVD + 2D CNN” based classifier which could effectively and reliably screen ULT classes. Our HW-SW device could be easily implemented on desktop assistive tool for individualized assessment of tremor progression, enabling self-care and medication adjustment requirements. This study was conducted under controlled experimental conditions using healthy subjects who simulated ULTs with the assistance of a metronome. The experimental results demonstrated the feasibility and effectiveness of the proposed mm-Wave sensor for classified controlled simulated tremor patterns, rather than providing direct clinical validation for PD diagnosis. Future work will involve clinically diagnosed PD patients as well as individuals with other tremor-related disorders to further evaluate the robustness and clinical applicability of the proposed method in real-world settings.

## Figures and Tables

**Table 1:** Feature extraction and enhancement with WVD and SPWVD processes for three ULT classes.

Frequency and ULT Class [7,30]	Feature Extraction	Feature Enhancement	
	mD Feature Pattern	WVD Processing	SPWVD Processing
Frequency: 0.0 Hz Resting Condition Normal	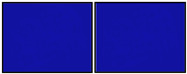	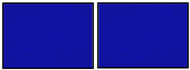	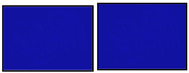
Low Frequency: <4.0 Hz Class I: Myorhythmia and Holmes Tremors	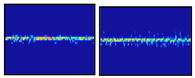	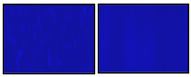	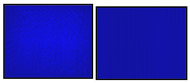
Medium Frequency: 4.0–7.0 Hz Class II: Parkinsonian Tremor (PDT), RT (4–6 Hz), PT (6–8 Hz)	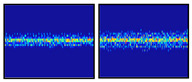	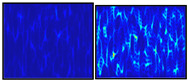	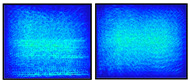
High Frequency: >7.0 Hz Class III: Essential Tremor (ET) PT (5–8 Hz), KT (5–12 Hz)	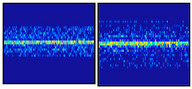	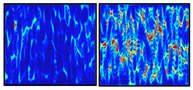	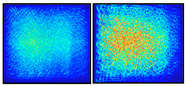

**Table 2:** Datasets at different measurement sites from 0.50 m to 1.50 m for three ULT classes.

Training and Testing Dataset		Measurement Site (m)			Feature Extraction Method
		0.50	1.00	1.50	
Class I	Training Dataset	600	600	600	micro-Doppler (mD) Features + WVD micro-Doppler (mD) Features + SPWVD
Class II		600	600	600	
Class III		600	600	600	
Class I	Testing Dataset	400	400	400	
Class II		400	400	400	
Class III		400	400	400	
Total		3000	3000	3000	

**Table 3:** Testing results of 10-fold cross-validation for “WVD + 2D CNN”, “SPWVD + 2D CNN”, “WVD + 1D CNN”, and “SPWVD + 1D CNN” based classifiers at the fixed 0.50 m measurement site.

Method	Average Loss Value	Average Precision (%)	Average Recall (%)	Average F1 Score	Average Accuracy (%)
WVD + 2D CNN	0.2309 ± 0.0020	93.86 ± 1.89	93.81 ± 1.88	0.9379 ± 0.1900	93.81 ± 1.88
SPWVD + 2D CNN	0.1244 ± 0.0012	95.92 ± 0.60	95.89 ± 0.62	0.9588 ± 0.0060	95.89 ± 0.62
WVD + 1D CNN	0.2798 ± 0.0020	90.35 ± 1.95	90.22 ± 2.01	0.9014 ± 0.0020	90.22 ± 2.01
SPWVD + 1D CNN	0.2942 ± 0.0020	88.25 ± 1.39	88.21 ± 1.37	0.8816 ± 0.0130	88.21 ± 1.37

**Table 4:** Testing results of 10-fold cross-validation for different classifier models and measurement sites.

Method	Site (m)	Average Loss Value	Average Precision (%)	Average Recall (%)	Average F1 Score	Average Accuracy (%)
mD Feature Pattern + 2D CNN	0.50	0.3422 ± 0.0484	88.61 ± 1.37	87.64 ± 1.41	0.8754 ± 0.0143	87.65 ± 1.41
	1.00	0.6157 ± 0.0365	74.42 ± 1.33	71.08 ± 1.91	0.7086 ± 0.0208	71.08 ± 1.91
	1.50	0.4654 ± 0.0156	80.43 ± 0.65	80.49 ± 0.56	0.7965 ± 0.0069	80.49 ± 0.56
WVD + 2D CNN	0.50	0.2309 ± 0.0020	93.86 ± 1.89	93.81 ± 1.88	0.9379 ± 0.1900	93.81 ± 1.88
	1.00	0.3126 ± 0.0030	88.25 ± 1.32	88.22 ± 1.30	0.8817 ± 0.1300	88.22 ± 1.30
	1.50	0.3903 ± 0.0030	85.42 ± 4.93	85.41 ± 4.67	0.8509 ± 0.0480	85.41 ± 4.67
SPWVD + 2D CNN	0.50	0.1244 ± 0.0012	95.92 ± 0.60	95.89 ± 0.62	0.9588 ± 0.0060	95.89 ± 0.62
	1.00	0.3816 ± 0.0030	87.39 ± 2.06	86.90 ± 1.93	0.8684 ± 0.1960	86.90 ± 1.93
	1.50	0.3357 ± 0.0030	85.98 ± 2.58	85.57 ± 2.27	0.8546 ± 0.0230	85.57 ± 2.27
WVD + 1D CNN	0.50	0.2798 ± 0.0020	90.35 ± 1.95	90.22 ± 2.01	0.9014 ± 0.0020	90.22 ± 2.01
	1.00	0.3855 ± 0.0030	83.57 ± 2.06	83.60 ± 2.06	0.8331 ± 0.0210	83.60 ± 2.06
	1.50	0.5077 ± 0.0040	79.87 ± 5.94	79.66 ± 5.93	0.7922 ± 0.0640	79.66 ± 5.93
SPWVD + 1D CNN	0.50	0.2942 ± 0.0020	88.25 ± 1.39	88.21 ± 1.37	0.8816 ± 0.0130	88.21 ± 1.37
	1.00	0.4439 ± 0.0040	80.66 ± 1.73	83.57 ± 1.12	0.8150 ± 0.1530	83.57 ± 1.12
	1.50	0.3216 ± 0.0030	81.51 ± 3.63	81.49 ± 3.51	0.8127 ± 0.0350	81.49 ± 3.51

**Table 5:** Comparisons of different non-contact and short-range sensing methods.

Sensing Method	Reference	Classification Method	Experimental Results
Digital Handwriting Method	[10]	Nonlinear SVM	Mean Hit Rate (%) = 92.13% for PD Screening
	[11]	GRNN	Mean Hit Rate (%) = 98.93% for PD Screening
	[23]	VGGs	Mean Accuracy (%) = 93.3% for PD Detection
		2D CNN	Mean Accuracy (%) = 93.3% for Early PD Diagnosis
X-band (10.525 GHz) Doppler mm-Wave Sensing with FMCW Short Range (0.2–0.4 m)	[7]	Linear Regression Method and Color Visual Representation	ULT Quantification: PDT (4–8 Hz), Myorhythmia and Holmes Tremors (<4 Hz), and Normal Condition ZC: R^2^ = 0.8949, WAMP: R^2^ = 0.8918, WL: R^2^ = 0.8553 for ULT Quantification
24 GHz FMCW MIMO Radar Non-contact Sensing in Short Range (0.7 m)	[49]	Lightweight CNN for 2D images processing and classification	Gesture Recognition: Wipe, Swing, Push, Invalid, and Circle Recognition Accuracy (%) = 99.60% for all Gesture Types
24 GH FMCW MIMO Radar (15 Virtual Channels) Non-contact Sensing Range (0–10 m)	[51]	3SF (Three-Step-Footprint) Algorithm + AA (Alternation Algorithm) Feet Identification Algorithm	Gait Symmetry Analysis: Gait Symmetry Ratio Errors: <8% (60 Subjects) Parameter: Step Time: 6%; Gait Velocity: 7% Stride Distance: 8%; Foot Velocity: 8%
W-band (77 GHz) Doppler mm-Wave Sensing with FMCW Non-contact Sensing in Short Range (3.0–6.0 m)	[29]	Linear Regression Method Correlation Plots for all Trials Tremor Frequency and Amplitude Parameters	Parkinson’s and Essential Tremor Quantification: Action, Posture, Rest Upper Limb, and Rest Lower Limb Tremors R^2^ > 0.966 for both Frequency and Amplitude. Parameters (Mean Values: 0.14 Hz and 0.03 cm)
Proposed Method (76–81 GHz) Non-contact Sensing in Short Range (0.5–1.0 m)	—	WVD + 2D CNN SPWVD + 2D CNN	ULT Classification:
			WVD + 2D CNN: Average Accuracy (%) = 93.81 ± 1.88%,
			Average F1 score = 0.9379 ± 0.1900 for ULT Classification
			SPWVD + 2D CNN: Average Accuracy (%) = 95.89 ± 0.62%,
			Average F1 score = 0.9588 ± 0.0060 for ULT Classification

## Data Availability

The dataset can be shared with the corresponding author upon request with justification.
